# The *IL7RA* rs6897932 polymorphism is associated with progression of liver fibrosis in patients with chronic hepatitis C: Repeated measurements design

**DOI:** 10.1371/journal.pone.0197115

**Published:** 2018-05-09

**Authors:** María Ángeles Jiménez-Sousa, Ana Zaida Gómez-Moreno, Daniel Pineda-Tenor, Luz Maria Medrano, Juan José Sánchez-Ruano, Amanda Fernández-Rodríguez, Tomas Artaza-Varasa, José Saura-Montalbán, Sonia Vázquez-Morón, Pablo Ryan, Salvador Resino

**Affiliations:** 1 Unidad de Infección Viral e Inmunidad, Centro Nacional de Microbiología, Instituto de Salud Carlos III, Majadahonda, Spain; 2 Servicio de Digestivo, Hospital Virgen de la Salud, Toledo, Spain; 3 Servicio de Laboratorio Clínico, Hospital Universitario de Fuenlabrada, Madrid, Spain; 4 Servicio de Laboratorio Clínico, Hospital Virgen de la Salud, Toledo, Spain; 5 Servicio de Medicina Interna, Hospital Universitario Infanta Leonor, Madrid, Spain; 6 Facultad de Medicina, Universidad Complutense de Madrid (UCM), Madrid, Spain; 7 Instituto de Investigación Sanitaria Gregorio Marañón (IiSGM), Madrid, Spain; Medizinische Fakultat der RWTH Aachen, GERMANY

## Abstract

The polymorphisms at the α-chain of the IL-7 receptor (*IL7RA*) have been related to T-cell homeostasis and development and may contribute to immune system deregulation. In the present study, we analyzed the association between *IL7RA* polymorphisms and the progression of liver fibrosis in patients infected with HCV. We carried out a retrospective study with a design consisting of repeated measurements in 187 HCV-infected patients, to study the risk prediction of liver fibrosis progression using genetic factors. We genotyped the rs6897932, rs987106 and rs3194051 *IL7RA* polymorphisms using the Agena Bioscience’s MassARRAY. Transient elastography was used to measure liver stiffness. The used cut-offs were: <7.1 kPa (F0-F1), 7.1–9.4 kPa (F2; significant fibrosis), 9.5-12.4 kPa (F3; advanced fibrosis), and ≥12.5 kPa (F4; cirrhosis). All HCV genotypes were analyzed. The median of follow-up time was 47.9 months. Baseline liver stiffness measurement (LSM) values did not show significant statistical differences for *IL7RA* genotypes (p>0.05). In univariate analysis, the rs6897932 T allele had a positive relationship with an increase in LSM (arithmetic mean ratio (AMR) = 1.21 (95%CI = 1.08; 1.36); p = 0.001), progression to advanced fibrosis (F≥3) (odds ratio (OR) = 2.51 (95%CI = 1.29; 4.88); p = 0.006) and progression to cirrhosis (F4) (OR = 2.71 (95%CI = 0.94; 5.03); p = 0.069). In multivariable analysis, the rs6897932 T allele was related to a higher increase of LSM values during follow-up (adjusted AMR = 1.27 (95%CI = 1.13; 1.42); p<0.001) and higher odds of progression to advanced fibrosis [adjusted OR = 4.46 (95%CI = 1.87; 10.62); p = 0.001], and progression to cirrhosis [adjusted OR = 3.92 (95%CI = 1.30; 11.77); p = 0.015]. Regarding *IL7RA* rs987106 and rs3194051 polymorphisms, we did not find significant results except for the relationship between *IL7RA* rs987106 and the increase in LSM values [adjusted OR = 1.12 (95%CI = 1.02; 1.23); p = 0.015]. The *IL7RA* rs6897932 polymorphism seems to be related to increased risk of liver fibrosis progression in HCV-infected patients. Thus, the rs6897932 polymorphism could be related to the physiopathology of CHC and might be used to successfully stratify the risk of CHC progression.

## Introduction

Hepatitis C virus (HCV) infection results in chronic hepatitis C (CHC) in about 80% of the subjects exposed. HCV infection is related to the development of liver fibrosis, cirrhosis and end-stage liver disease [1–3], and is a major cause for liver transplantation [4,5]. However, CHC progression presents a great deal of variability, from reduced rates of liver fibrosis progression to massive fibrosis and cirrhosis in a short time [6]. The clinical management of CHC patients depends on the disease stage [7], since patients with cirrhosis have a shorter median survival time than those without cirrhosis [6]. Thus, the appropriate identification of patients at risk of liver fibrosis progression and cirrhosis is essential to take preventive measures that may affect the course of CHC [8]. In this regard, transient elastography may accurately predict the presence of fibrosis/cirrhosis in patients with CHC [9]. However, the recent introduction of direct-acting antivirals (DAAs) has revolutionized HCV treatment with sustained virological response rates greater than 90% [10]. HCV eradication has been associated with a reduction in the risk of liver-related events such as hepatic decompensation, hepatocellular carcinoma and death due to liver disease [11]. However, patients with fibrosis bridges (F3) or cirrhosis (F4), despite the eradication of HCV, remain at risk of progression of the disease and of hepatocellular carcinoma at least 8–10 years after the virological cure [12,13].

Interleukin-7 (IL-7) plays a role in homeostasis and development of T cells [14]. IL-7 is also a critical factor in T-cell-mediated antiviral response [15]. The IL-7 receptor is made up of a heterodimer consisting of the α-chain of the IL-7 receptor (IL7RA or CD127) and the gamma chain of the common cytokine receptor (CD132) [14]. In this way, HCV clearance is related to the early expression of IL7RA on HCV-specific T cells during the acute phase of HCV infection [16]. Additionally, IL-7 released by hepatocytes during HCV infection eventually leads to viral clearance [17]. Moreover, IL-7 and IL7RA levels decrease during CHC, which leads to impaired reactivity of HCV-specific cytotoxic T cells [18,19].

*IL7RA* polymorphisms have been related to the homeostasis and development of T-cells, so they may contribute to the deregulation of the immune system [20,21]. Furthermore, *IL7RA* polymorphisms are related to autoimmune diseases [22,23] and CD4+ T cell recovery in human immunodeficiency virus (HIV)-infected patients on antiretroviral therapy [24–26]. Among HCV-infected patients, *IL7RA* polymorphisms have been associated with the response to peg-interferon (IFN)-α/ribavirin therapy against HCV and severe liver fibrosis in HIV/HCV-coinfected patients [27,28].

We aimed to analyze the relationship between *IL7RA* polymorphisms and the progression of liver fibrosis in patients infected with HCV in a study with repeated measurements.

## Materials and methods

### Study population

We performed a retrospective cohort study (repeated measurements design) in 187 HCV-infected patients in the Hospital Virgen de la Salud (Toledo, Spain) between 2008 and 2015. This study was developed according to the ethical principles of the 1975 Declaration of Helsinki. The study was approved by the Institutional Review Board of the Instituto de Salud Carlos III (“Comités de Ética de la Investigación y Bienestar Animal”– 04/04/2013). Patients included in this study signed a written informed consent. This study has been reported in accordance with the FibroSTARD guidelines.

We considered as inclusion criteria: 1) detectable HCV RNA in plasma during follow-up; 2) data availability of liver stiffness measurement (LSM) for a range of at least 12 months; 3) availability of a DNA sample. The exclusion criteria were: 1) advanced fibrosis or cirrhosis at baseline (F3 or F4; LSM≥9.5); 2) co-infection with hepatitis B virus or HIV; 3) autoimmune liver disease such as primary biliary cholangitis and autoimmune hepatitis.

**Fig 1:**
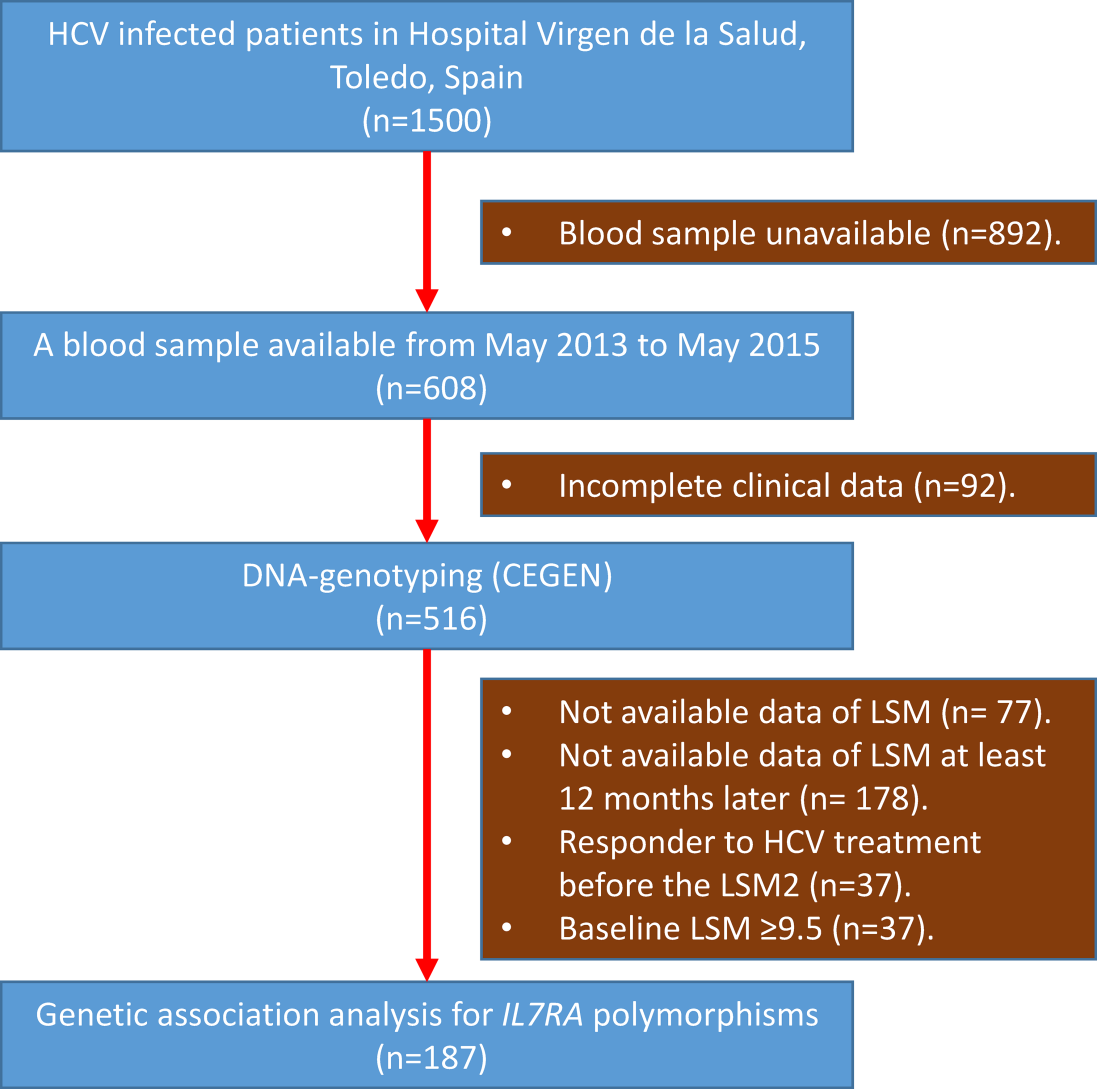
Flow chart of patients included in the genetic association analysis for *IL7RA* polymorphisms. Abbreviations: HCV, hepatitis C virus; *IL7RA*, gene of α-chain of the IL-7 receptor; LSM, liver stiffness measurement (LSM).

From around 1500 HCV-infected patients attended to at the Hospital Virgen de la Salud (Toledo, Spain), we had 608 blood samples available from May 2013 to May 2015. Of them, 92 patients were discarded due to incomplete clinical data, leaving 516 patient samples that were sent for DNA genotyping. These 516 patients constitute our study cohort for this and future studies. Of them, 77 patients were discarded due to unavailable LSM data, 178 patients due to unavailable LSM2 data at least 12 months after baseline LSM, 37 patients for being responders to HCV treatment before the last LSM, and 37 patients because the baseline LSM was ≥9.5 (**Fig 1**).

### Clinical data

Medical records were used to obtain clinical and epidemiological data. We considered a high alcohol intake to be levels above 60 grams/day for men and above 20 grams/day for women, which has been described as a risk factor for the development of cirrhosis [29]. Time since HCV diagnosis was defined as the time between HCV diagnosis and the first LSM (LSM1, the baseline of study).

The management of the patients during follow-up was conventional. For this reason, HCV therapy could be used before or after the inclusion into the study, following the recommendations of clinical guidelines [30,31]. However, we only included those patients who were non-responders from patients undergoing HCV treatment. In the case of patients who achieved a sustained virological response (SVR), their follow-ups were truncated at the time of HCV therapy initiation, because elimination of HCV may cause the regression of liver fibrosis and cirrhosis [32]. The time of follow-up was the time between the first LSM (LSM1) and the last LSM (LSM2, the end of study). The administrative censoring date was March 31, 2016.

### Genotyping of *IL7RA* polymorphisms

Three single nucleotide polymorphisms (SNPs) located at the *IL7RA* gene [rs987106 (intronic region), rs6897932 (exon 6) and rs3194051 (exon 8)] were evaluated in all patients who met the inclusion criteria regardless of LSM value. Our group has previously analyzed these polymorphisms in HIV-coinfected patients in two recent papers [27,28]. These *IL7RA* polymorphisms are located at a putative regulatory region, but their effects still are unclear. Their minor allelic frequencies were greater than 20% in white Europeans.

QIAsymphony DNA Mini Kit (Qiagen, Hilden, Germany) was used to extract DNA from 200μl of peripheral blood. The Spanish National Genotyping Center (CeGen; http://www.cegen.org/) genotyped the samples by Agena Bioscience’s MassARRAY platform (San Diego, CA, USA) using the iPLEX® Gold assay design system.

The 1000 Genomes Project website (http://www.1000genomes.org/home) were used to obtain the frequencies of alleles and genotypes represented in the general population. This database contains a broad representation of common human genetic variation from multiple populations [33], including the Iberian population in Spain (IBS) and Utah residents with Northern and Western European ancestry (CEU), which were included in the study as a comparative control.

### Liver stiffness measurement

One trained hepatologist evaluated liver stiffness using a single machine of transient elastography (FibroScan®, Echosens, Paris, France). The results were expressed in kilopascals (kPa), with a range of 2.5 to 75 kPa [34]. We considered the measurements appropriate when the interquartile-range-to-median ratio for at least ten successful measurements was lower than 0.30. All LSM measurements were performed with at least four hours of fasting. Advanced obese patients were not included in this study because we did not have access to an XL probe, and the M probe was used in all cases. The cut-offs used were: <7.1 kPa (F0-F1), 7.1–9.4 kPa (F2; significant fibrosis), 9.5-12.4 kPa (F3; advanced fibrosis), and ≥12.5 kPa (F4; cirrhosis) [35]. In this regard, many LSM cut-offs have been reported recently for stratifying patients according to liver fibrosis. However, we used the LSM cut-offs described by Castera et al (2005) [35], because these have been validated by a large number of scientific articles and have been recently endorsed by the American Gastroenterological Association [36].

### Outcome variable

The primary outcome variable was the change in LSM values during follow-up (continuous variable). We calculated the LSM variation between the last LSM (LSM2) and the first LSM (LSM1) during follow-up (ratio LSM2/LSM1). Furthermore, we evaluated the progression to advanced fibrosis, which is a dichotomous variable that may have values of +1 [if F≤2 (F0, F1 or F2) changes to F≥3 (F3 or F4)] or 0 [if F ≤2 (F0, F1 or F2) remains)]. The progression to cirrhosis may also have values of +1 [if F≤2 (F0, F1, or F2) changes to F4] or 0 (if F≤2 (F0, F1 or F2) remains).

### Statistical analysis

We evaluated the genetic association according to dominant, recessive and additive models of inheritance. The best model was chosen according to the goodness of fit evaluated by Akaike information criterion (AIC) value and Bayesian information criterion (BIC).

The Chi-square test or Fisher’s exact test (categorical variables) and Mann-Whitney U test (continuous variables) were used to evaluate the differences between *IL7RA* genotypes. The genetic association of *IL7RA* polymorphisms with the outcome variables was evaluated using a Generalized Linear Model (GLM). When we analyzed continuous variables, a GLM with a gamma distribution (log-link) was employed. This test provides the differences between groups and the arithmetic mean ratio (AMR) that indicates the ratio by which the arithmetic mean of the original outcome should be multiplied. In this way, a GLM with a binomial distribution (logit-link) was employed to investigate dichotomous outcome variables (progression to F≥3 and progression to F4). This test provides the odds ratio (OR) for dichotomous outcome variables. Each multivariable analysis was adjusted by the most significant co-variables associated with each one of the outcome variables, avoiding the over-fitting of the model. At each step, covariables were considered for removal with a p-value for exit greater than 0.50. The factors used were age, gender, diabetes, time since HCV diagnosis, HCV genotype, injection drug use (IDU), high alcohol intake, HCV antiviral therapy before baseline and during follow-up (patients who failed therapy), the baseline of LSM, and the time of follow-up. Although IDU does not have a direct association with liver fibrosis progression, we included IDU as a covariable because these subjects generally have a worse lifestyle, which can negatively affect the progression of liver disease [36].

The Statistical Package for the Social Sciences (SPSS) 21.0 software (IBM Corp., Chicago, USA) was used to perform the statistical tests. Haploview 4.2 software was used to evaluate the pairwise linkage disequilibrium (LD). The frequencies of haplotypes were estimated considering the Expectation-Maximization algorithm. PLINK software (http://zzz.bwh.harvard.edu/plink/) was used to analyze the association between haplotypes and fibrosis progression. All p-values were two-tailed and statistical significance was defined as p<0.05.

## Results

### Study population

The baseline characteristics of 187 HCV-infected patients are summarized in **Table 1**.

**Table 1:** Clinical and epidemiological characteristics of HCV-infected patients.

Characteristic	All Patients
**No.**	187
**Male sex**	102 (54.5%)
**Age (years)**	46.4 (40.9; 55.8)
**Time of HCV infection (years)**	7.5 (2.9; 12.9)
**High alcohol intake**	25 (13.4%)
**Prior injection drug use**	20 (10.7%)
**HCV genotype (n = 184)**	
**1**	154 (83.7%)
**3**	14 (7.6%)
**4**	15 (8.2%)
**5**	1 (0.5%)
**Prior peg-IFN-α/RBV therapy failed**	42 (22.5%)
**Baseline LSM (kPa)**	6.1 (5.3; 8.4)
**F0-F1 (<7.1 kPa)**	149 (79.7%)
**F2 (7.1–9.4 kPa)**	38 (20.3%)
**Follow-up time (months)**	47.9 (29.2; 62.7)
**Final LSM (kPa)**	6.7 (5.3; 8.6)
**F0-F1 (<7.1 kPa)**	108 (57.8%)
**F2 (7.1–9.4 kPa)**	47 (25.1%)
**F3 (9.5–12.4 kPa)**	15 (8.0%)
**F4 (≥12.5 kPa)**	17 (9.1%)

Values expressed as absolute numbers (%) and median (percentile 25; percentile 75). High alcohol intake was considered as levels above 60 grams/day for men and above 20 grams/day for women.

**Abbreviations:** Abbreviations: HCV, hepatitis C virus; LSM, liver stiffness measurement; kPa, kilopascal; IFN, interferon; peg-IFN-α/RBV, peg-interferon-alpha/ribavirin.

We observed that 38 patients presented significant fibrosis (LSM≥7.1 kPa). Male patients made up 54.5% of the total, and the median age was 46.4 years, with a high intake of alcohol rate of 13.4%. Nearly eleven percent of patients were prior injection drug users, 83.7% were infected with HCV genotype 1, and 22.5% had previously failed antiviral therapy (peg-IFN-α/ribavirin). The median LSM in the baseline was 6.1 kPa and the follow-up time was 47.9 months.

### Characteristics of *IL7RA* polymorphisms

**Fig 2:**
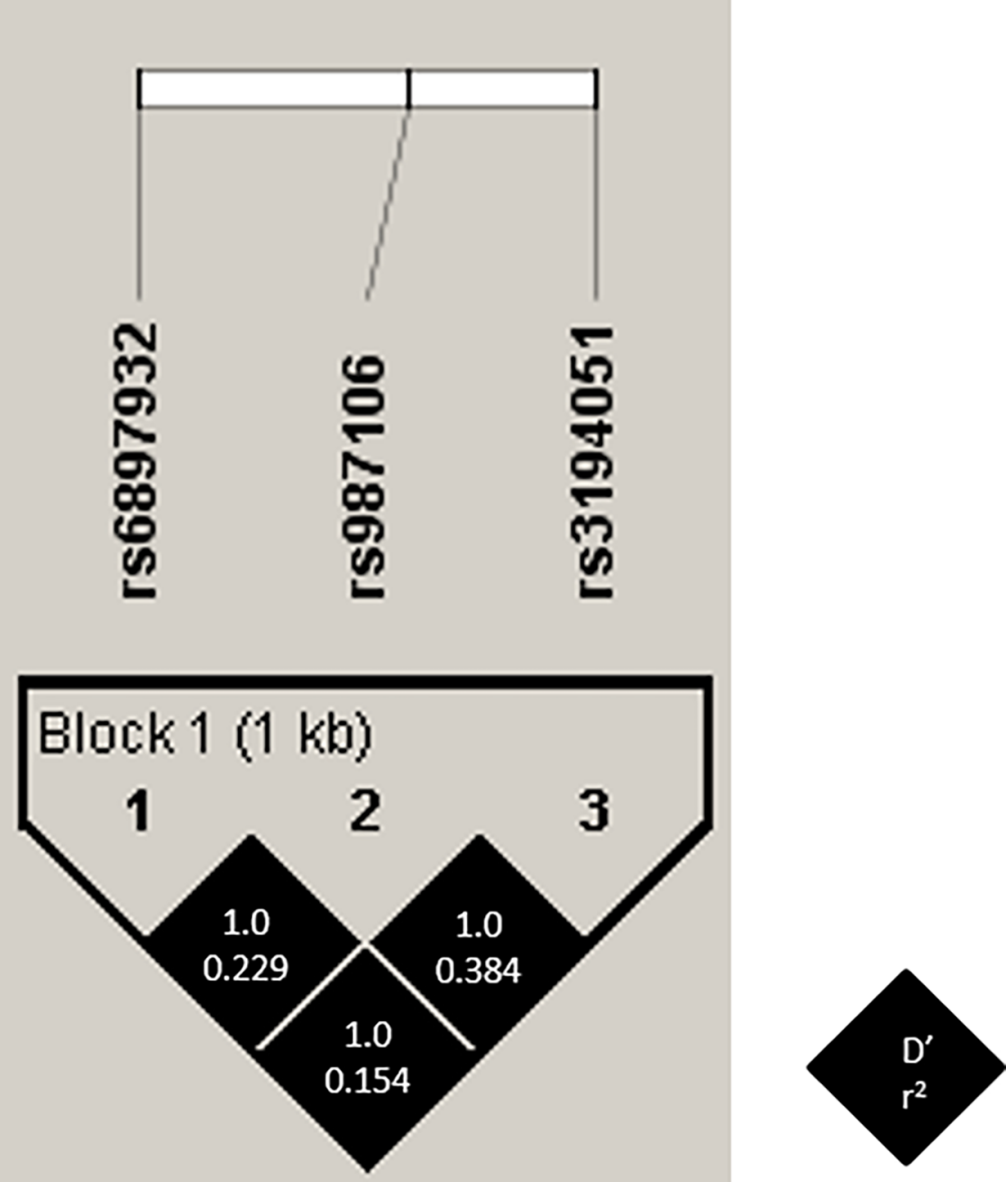
Pairwise linkage disequilibrium (LD) pattern for *IL7RA* polymorphisms. Each diagonal represents a different SNP, with each square representing a pairwise comparison between two SNPs.

The *IL7RA* polymorphisms showed a strong linkage disequilibrium (LD) (non-random association of alleles at different loci) (D´ = 1.0). Nevertheless, the r-square among SNPs was low (r-square<0.40) (**Fig 2**).

The allelic and genotypic frequencies of the *IL7RA* polymorphisms are shown in **Table 2.** These frequencies were compared to those for healthy subjects extracted from the 1000 genomes database, and no significant differences between groups were found. All SNPs had a minimum allele frequency >5% and were in Hardy-Weinberg equilibrium (p>0.05). We did not obtain any missing value for any *IL7RA* polymorphisms.

**Table 2:** Summary of Hardy Weinberg Equilibrium and frequencies of alleles and genotypes for *IL7RA* polymorphisms in HCV-infected patients compared to Iberian populations in Spain (IBS) and Utah residents with Northern and Western European ancestry (CEU) from 1000 genomes project data (http://browser.1000genomes.org/index.html).

	HCV group (n = 187)					IBS group (n = 107)					χ^2^ test(a)	χ^2^ test (b)	CEU group (n = 99)					χ^2^ test(a)	χ^2^ test (b)
SNPs	HWE	Alleles		Genotypes		HWE	Alleles		Genotypes		p-value	p-value	HWE	Alleles		Genotypes		p-value	p-value
**rs6897932**	0.216	C	76.7%	CC	56.7%	0.551	C	74.8%	CC	57.0%	0.714	0.225	0.507	C	75.8%	CC	58.6%	0.865	0.255
		T	23.3%	CT	40.1%		T	25.2%	CT	35.5%				T	24.2%	CT	34.3%		
				TT	3.2%				TT	7.5%						TT	7.1%		
**rs987106**	0.605	A	43.0%	AA	19.8%	0.308	A	49.5%	AA	27.1%	0.283	0.308	0.456	A	42.4%	AA	16.2%	0.922	0.592
		T	57.0%	AT	46.5%		T	50.5%	AT	44.9%				T	57.6%	AT	52.5%		
				TT	33.7%				TT	28.0%						TT	31.3%		
**rs3194051**	0.255	A	66.3%	AA	46.5%	0.937	A	74.8%	AA	56.1%	0.130	0.100	0.646	A	66.7%	AA	43.4%	0.946	0.446
		G	33.7%	AG	39.6%		G	25.2%	AG	37.4%				G	33.3%	AG	46.5%		
				GG	13.9%				GG	6.5%						GG	10.1%		

**Statistical:** p-values were calculated by Chi-squared test; (a), differences between allele frequencies; (b), differences between genotype frequencies.

**Abbreviations**: HWE, Hardy Weinberg Equilibrium; HCV, hepatitis C virus; IL7RA, α-chain of the interleukin 7 receptor.

### *IL7RA* polymorphisms and liver fibrosis progression

Baseline LSM values did not show significant differences among *IL7RA* genotypes (p>0.05). We only obtained significant results for the *IL7RA* rs6897932 polymorphism. Additionally, the additive model was the one that best fit our data.

**Fig 3:**
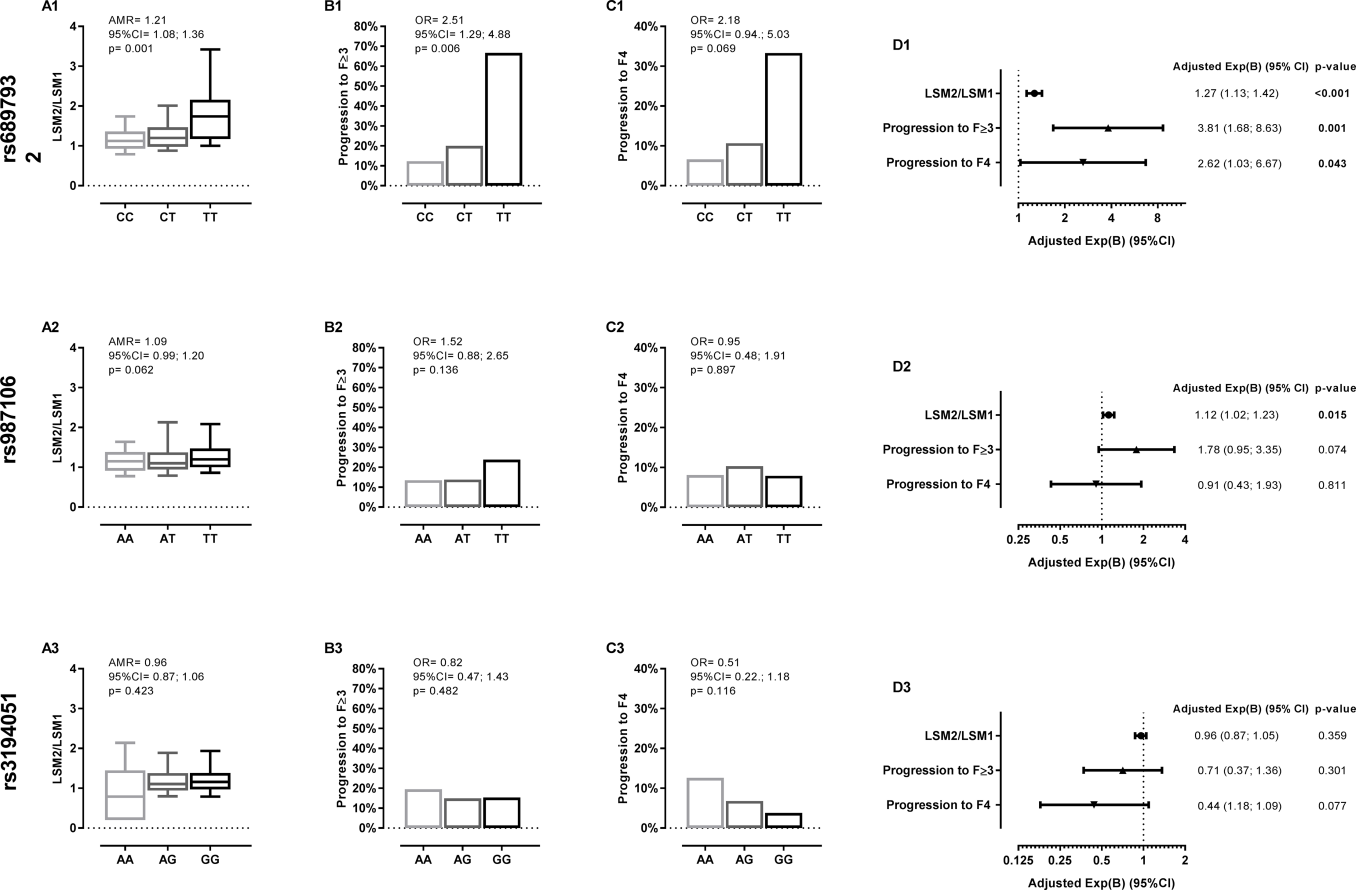
Summary of the relationship between *IL7RA* polymorphisms and variation of liver stiffness measurements (LSM) and fibrosis stages in HCV-infected patients with an additive inheritance model. **Statistical**: p-values were calculated by univariate and multivariable regression adjusted by the most important clinical and epidemiological characteristics (see statistical analysis section). Significant differences are shown in bold. **Abbreviations**: LSM, liver stiffness measure; kPa, kilopascal; LSM1, baseline LSM; LSM2, final LSM; Exp(B), arithmetic mean ratio (AMR) for continuous variable and odds ratio (OR) for categorical variables; 95%CI, 95% of confidence interval; p-value, level of significance; F≥3, advanced fibrosis; F4, cirrhosis; IL7RA, α-chain of the interleukin 7 receptor.

In univariate analysis, the rs6897932 T allele had a significant positive relationship with an increase in LSM values (AMR = 1.21 (95%CI = 1.08; 1.36); p = 0.001; **Fig 3A1**), progression to advanced fibrosis (OR = 2.51 (95%CI = 1.29; 4.88); p = 0.006; **Fig 3B1**) and progression to cirrhosis (OR = 2.71 (95%CI = 0.94; 5.03); p = 0.069; **Fig 3C1**). These trends are maintained in the multivariable analysis adjusted by the main clinical and epidemiological covariates (**Fig 3D1**). The rs6897932 T allele was related to a higher increase in LSM values during follow-up (adjusted AMR = 1.27 (95%CI = 1.13; 1.42); p<0.001), and higher odds of progression to advanced fibrosis [adjusted OR = 4.46 (95%CI = 1.87; 10.62); p = 0.001], and progression to cirrhosis [adjusted OR = 3.92 (95%CI = 1.30; 11.77); p = 0.015] (**Fig 3D1**).

In analysis of the *IL7RA* rs987106 and rs3194051 polymorphisms, we did not find significant results except for the relationship between *IL7RA* rs987106 and the increase in LSM values [adjusted OR = 1.12 (95%CI = 1.02; 1.23); p = 0.015] (**Fig 3D2**). In addition, **S1 Fig** shows data for all *IL7RA* polymorphisms according to recessive and dominant models.

## Discussion

In this study, we stratified our HCV-infected patients by *IL7RA* genotypes, and we evaluated the progression of liver fibrosis, which constitutes a critical element of the management of patients with CHC by providing a useful prognostic tool and helping in treatment decisions [7,37]. The major finding of our study was that the rs6897932 T allele was associated with a higher risk of liver fibrosis progression in HCV-infected patients. However, in our study rs987106 showed a weaker association with liver disease progression and rs3194051 showed none. We must emphasize that in addition to the additive model we also found a significant association with liver fibrosis progression when we used the recessive and dominant models, but with a slightly lower statistical significance. To our knowledge, this is the first article that relates *IL7RA* polymorphisms with liver fibrosis progression in HCV-monoinfected patients. Additionally, our study has a longitudinal design of repeated measurements with a follow-up over a prolonged period of time. This dynamic design is more valid for detecting changes at the individual level, which is a novel aspect of our results in comparison with previous findings.

The *IL7RA* rs6897932 polymorphism constitutes a replacement of a threonine by an isoleucine in the IL7RA (also called CD127) transmembrane domain. This fact could affect the levels of the soluble IL7RA isoform (sIL7RA) and membrane-bound IL7RA isoform by putatively disrupting an exonic splicing motif [22]. The rs6897932 T allele, in contrast to the rs6897932 C allele, has been associated with lower plasma levels of sIL7RA [22,38–40], without limiting the effect of circulating IL-7 [39,41]. By contrast, the rs6897932 C allele has been related to elevated plasma concentrations of sIL7RA [22,38–40], which is able to bind to circulating IL-7 and reduce the bioavailability of IL-7, limiting its effects [39,41]. Moreover, it has been described that the “TT” genotype has increased signal transduction and proliferation in response to IL-7 among HIV-infected individuals [42]. This fact may explain the positive effect of the rs6897932 T allele on CD4+ T cell recovery in HIV-infected patients on antiretroviral therapy [24,25,40]. In our study, patients with the rs6897932 T allele had a higher risk of liver fibrosis progression during follow-up. Therefore, it would be plausible that the rs6897932 T allele, versus C allele, may confer lower levels of sIL7RA, higher IL-7 bioavailability [41] and higher immune response against HCV infection [17]. Thus, it may lead to a greater destruction of hepatocytes and liver injury, which favors the progression of liver fibrosis during CHC [43].

Moreover, in a previous cross-sectional study, we observed that both rs987106 and rs3194051 polymorphisms were associated with advanced fibrosis in HIV/HCV-coinfected patients [28]. However, in the current study, we found a weak association between rs987106 and liver disease progression and none for rs3194051 polymorphism. The discrepancy observed for these two SNPs may be due to multiple reasons, among which we should highlight the study design (cross-sectional vs. longitudinal) and the type of patients included (HIV/HCV-coinfected vs. HCV-monoinfected). Moreover, in our study, a high LD was observed, but the r-square among SNPs was low (r-square<0.40), meaning that the *IL7RA* polymorphisms provide different information and cannot be substituted one for the other, supporting the differential association observed among SNPs in this study.

From now going forward with the new DAAs to treat HCV infection, all HCV-infected patients will be treated in the short or medium term, resulting in a virological cure for 90–95% of them. However, it has recently been reported that disease progression and the incidence of hepatocellular carcinoma have both increased in patients who achieved SVR [44–47]. Under this new context, further studies will be needed to investigate whether the association between the rs6897932 T allele and fibrosis progression is maintained after SVR, which could shed light on the importance of the *IL7RA* polymorphism on the clinical evolution of patients with HCV clearance.

This study has several limitations that must be considered. Firstly, we used a retrospective design that included patients who came to the hospital and had a sufficient follow-up. However, the repeated measurements design provides robustness to this study. Secondly, the number of samples was small and the reduced number of patients with some *IL7RA* genotypes may have made the detection of associations more difficult, as well as, the accuracy of the risk estimation. Therefore, independent replication studies with a larger sample size, including different ethnic groups, may be necessary. Thirdly, we did not have access to plasma specimens, so the concentration in plasma was missing for both IL-7 and sIL7RA. Fourthly, we performed the analysis on European patients only, and studies would benefit if they were performed on different ethnic groups. Fifthly, around 23% of patients were previously treated with IFN therapy, but they maintained the HCV infection. This treatment failure does not affect the natural course of CHC during long-term follow up [48,49], so these non-responders were not excluded. Moreover, we did not find any significant effect of IFN-failed therapy on the statistical analysis of liver fibrosis progression. Sixthly, we could not study the association between *IL7RA* polymorphisms and hepatocellular carcinoma because only a few patients developed hepatocellular carcinoma in our cohort. Additionally, we did not have enough data of some relevant clinical variables (e.g., HCV viral load, ALT levels), which were therefore not considered in the multivariable analysis. Finally, regarding multiple testing, there is a considerable controversy about adjusting the “p-value” in clinical-orientated studies ^24,25^. In our case, we were not literally doing a random search for a meaningful result because we have a hypothesis supported by theory and previous articles that have shown relationships of *IL7RA* polymorphisms to viral infection outcomes [24–26], including HCV infection [27,28]. Thus, our results should not be affected by carrying out a high number of statistical tests.

## Conclusions

In conclusion, the *IL7RA* rs6897932 polymorphism seems to be related to increased risk of liver fibrosis progression in HCV-infected patients. Thus, the rs6897932 polymorphism could play a crucial role in the physiopathology of CHC and could be used to stratify the risk of CHC progression.

## Supporting information

S1 Fig Summary of the relationship between *IL7RA* polymorphisms and variation of liver stiffness measurements (LSM) and fibrosis stages in HCV-infected patients with recessive and dominant inheritance model. **Statistical**: p-values were calculated by multivariable regression adjusted by the most important clinical and epidemiological characteristics (see statistical analysis section). Significant differences are shown in bold.
**Abbreviations**: LSM, liver stiffness measure; LSM1, baseline LSM; LSM2, final LSM; Exp(B), arithmetic mean ratio (AMR) for continuous variable and odds ratio (OR) for categorical variables; 95%CI, 95% of confidence interval; p-value, level of significance; F≥3, advanced fibrosis; F4, cirrhosis; IL7RA, α-chain of the interleukin 7 receptor.
(TIF)
